# MicroRNA-210 Suppresses Junction Proteins and Disrupts Blood-Brain Barrier Integrity in Neonatal Rat Hypoxic-Ischemic Brain Injury

**DOI:** 10.3390/ijms18071356

**Published:** 2017-06-24

**Authors:** Qingyi Ma, Chiranjib Dasgupta, Yong Li, Lei Huang, Lubo Zhang

**Affiliations:** Center for Neonatal Biology, Division of Pharmacology, Department of Basic Sciences, School of Medicine, Loma Linda University, Loma Linda, CA 92350, USA; cdasgupta@llu.edu (C.D.); yoli@llu.edu (Y.L.); lehuang@llu.edu (L.H.); lzhang@llu.edu (L.Z.)

**Keywords:** neonatal hypoxic-ischemic brain injury, microRNA-210, blood-brain barrier integrity, cerebral edema

## Abstract

Cerebral edema, primarily caused by disruption of the blood-brain barrier (BBB), is one of the serious complications associated with brain injury in neonatal hypoxic-ischemic encephalopathy (HIE). Our recent study demonstrated that the hypoxic-ischemic (HI) treatment significantly increased microRNA-210 (miR-210) in the neonatal rat brain and inhibition of miR-210 provided neuroprotection in neonatal HI brain injury. The present study aims to determine the role of miR-210 in the regulation of BBB integrity in the developing brain. miR-210 mimic was administered via intracerebroventricular injection (i.c.v.) into the brain of rat pups. Forty-eight hours after the injection, a modified Rice-Vannucci model was conducted to produce HI brain injury. Post-assays included cerebral edema analysis, western blotting, and immunofluorescence staining for serum immunoglobulin G (IgG) leakage. The results showed that miR-210 mimic exacerbated cerebral edema and IgG leakage into the brain parenchyma. In contrast, inhibition of miR-210 with its complementary locked nucleic acid oligonucleotides (miR-210-LNA) significantly reduced cerebral edema and IgG leakage. These findings suggest that miR-210 negatively regulates BBB integrity in the neonatal brain. Mechanistically, the seed sequences of miR-210 were identified complementary to the 3′ untranslated region (3′ UTR) of the mRNA transcripts of tight junction protein occludin and adherens junction protein β-catenin, indicating downstream targets of miR-210. This was further validated by in vivo data showing that miR-210 mimic significantly reduced the expression of these junction proteins in rat pup brains. Of importance, miR-210-LNA preserved the expression of junction proteins occludin and β-catenin from neonatal HI insult. Altogether, the present study reveals a novel mechanism of miR-210 in impairing BBB integrity that contributes to cerebral edema formation after neonatal HI insult, and provides new insights in miR-210-LNA mediated neuroprotection in neonatal HI brain injury.

## 1. Introduction

Perinatal hypoxic-ischemic encephalopathy (HIE) is the leading cause of acute mortality and morbidity in newborns with an incidence of one to eight cases per 1000 term births. Survivors often suffer from cognitive impairment, seizures, and motor impairment (cerebral palsy) [1,2,3]. The molecular mechanisms of brain injury in infants with HIE remain largely elusive. Only hypothermia treatment has currently been proven to provide some degree of clinical success [4,5]. The better understanding of the pathogenesis of neonatal HIE is crucial to develop novel and effective treatment plans for neonatal HI brain injury.

Cerebral edema is primarily caused by breakdown of the blood-brain barrier (BBB) junction complexes, and is one of the important causes in neonatal hypoxic-ischemic (HI) brain injury and brain tissue damage [6,7,8]. It has been documented that up to 86% of term neonates who suffer from severe neonatal asphyxia develop cerebral edema [9], which is correlated to poor neurological outcomes [10]. Animal studies using magnetic resonance imaging have shown that the development of cerebral edema starts several hours after HI insult, reaches the peak at 24–48 h, and then begins to be resolved [11,12]. Mounting evidence indicates that uncontrolled increase in BBB permeability is the hallmark of multiple neurological diseases [13,14], including neonatal HIE [15,16,17,18]. Indeed, the cerebral microvascular response a nd BBB damage occur rapidly and are primarily responsible for the brain injury observed in neonatal HI model [15,16]. The current evidence strongly suggests that BBB damage is one of the major therapy targets for neonatal HI brain injury. The BBB is a highly specialized endothelial structure that acts as a biochemical barrier, separating brain tissues from the substances circulating in the blood and maintaining the homeostasis of the brain microenvironment. The barrier property of the BBB is determined by junction complexes between cerebral endothelial cells, including tight junctions (TJs), which consist of occludin, claudins, zonula occludens protein-1 (ZO-1), etc. [19,20], and adherens junctions (AJs), a complex that consists of vascular endothelial cadherin (VE-cadherin), α-catenin, β-catenin, etc. [21,22]. The disruption of junction proteins leads to increased paracellular permeability of the brain endothelial barrier in pathological conditions [16,23,24], indicating the regulation of junction proteins as an important therapeutic target for HI brain injury.

Growing evidence has revealed that microRNAs (miRs) are involved in various neurological disorders including ischemic stroke [25,26]. The mature miRs are composed of about 21–22 nucleotides, which usually repress the expression of genes by binding to the 3′ untranslated region (3′ UTR) of target mRNAs [27,28]. Among them, miR-210 is considered as the *Master Hypoxamir* of a specific group of miRs that responds to hypoxia in multiple cell types [29,30]. For instance, miR-210 plays important roles in the regulation of endothelial cell survival [30,31] and vascular angiogenesis in response to hypoxia [32]. Recently, we demonstrated that the HI treatment significantly increased miR-210 in the neonatal rat brain and inhibition of miR-210 provided neuroprotection in neonatal HI brain injury [33]. However, it remains undetermined whether and to what extent miR-210 participates in the regulation of the BBB disruption in neonatal HI brain injury.

Herein, we present evidence of a novel mechanism that miR-210 impairs the BBB integrity by suppressing the expression of junction proteins, thus resulting in increased susceptibility of the BBB to HI insult in neonatal rats. Of importance, the inhibition of miR-210 with complementary locked nucleic acid (LNA) oligonucleotides 4 h after brain HI insult increases the abundance of junction proteins in the brain, and significantly reduces BBB leakage and cerebral edema, suggesting a novel mechanism of the neuroprotective effect mediated by miR-210 inhibition on neonatal HIE.

## 2. Results

### 2.1. miR-210 Mimic Exacerbates Cerebral Edema and Immunoglobulin G (IgG) Leakage Following Neonatal HI Insult

**Figure 1:**
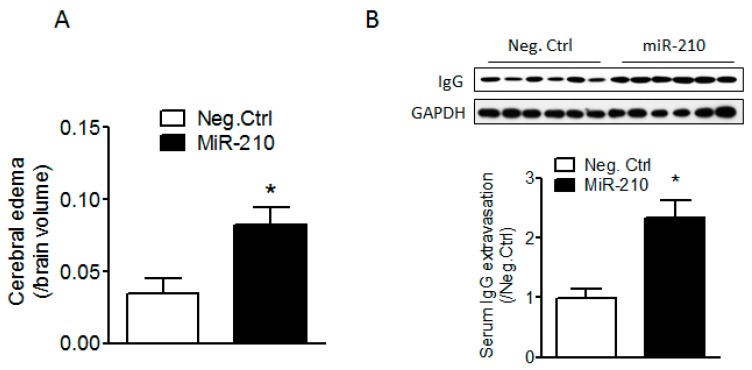
miR-210 exacerbates cerebral edema and serum immunoglobulin G (IgG) leakage after neonatal hypoxic-ischemic (HI) insult. Pups received either miR-210 mimic or its negative control (Neg. Ctrl) via intracerebroventricular injection (i.c.v.) injection 48 h prior to HI insult (1.5 h of hypoxia, 8% O_2_). Cerebral edema (**A**) was determined 48 h following HI based on the infarct volume assay, and serum IgG extravasation into the brain tissue (**B**) was determined 24 h following HI by western blotting. *n* = 6–8. * *p* < 0.05, miR-210 vs. Neg. Ctrl.

To explore whether miR-210 increased the vulnerability of the BBB in neonatal brain to HI insults, either miR-210 mimic or its negative control was delivered into the brain of rat pups through intracerebroventricular (i.c.v.) injection. HI brain injury was produced 48 h after injection. Cerebral edema volumes were calculated by subtracting indirect from direct infarct volumes as previously described [34,35]. Our results showed that miR-210 mimic significantly increased cerebral edema 48 h (Figure 1A) and serum IgG leakage into brain tissue 24 h (Figure 1B) after HI as compared to its negative control, which suggests that miR-210 increases BBB injury in the neonatal brain in response to HI insult.

### 2.2. miR-210 Inhibition Reduces Cerebral Edema and Serum IgG Leakage Following Neonatal HI

**Figure 2:**
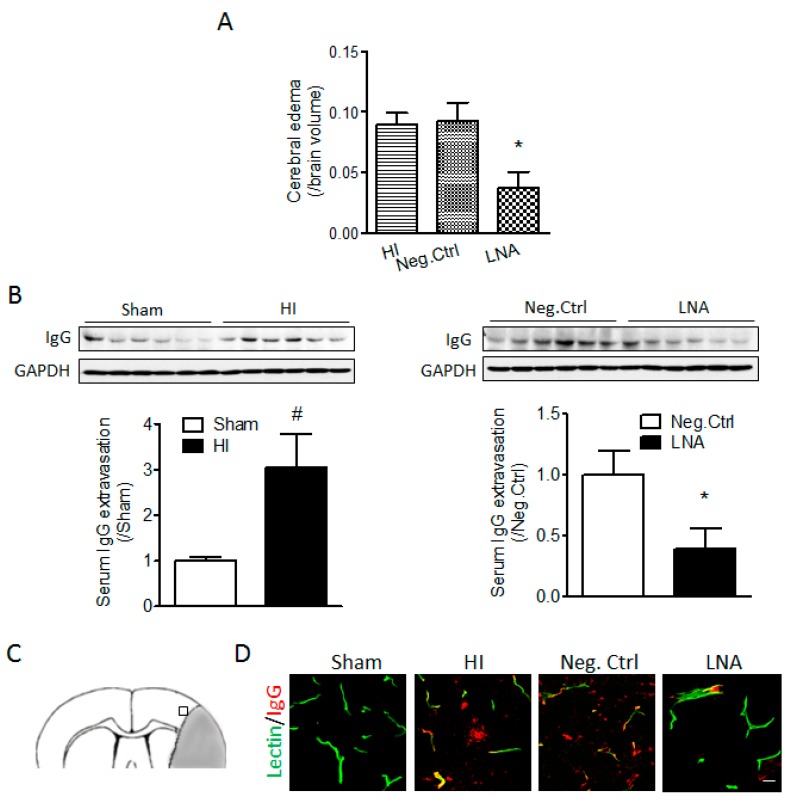
miR-210-LNA treatment reduces cerebral edema and serum IgG leakage after neonatal HI insult. Pups received either miR-210-LNA or its negative control (Neg. Ctrl) via i.c.v. injection 4 h after HI insult (2.0 h of hypoxia, 8% O_2_). Cerebral edema (**A**) was determined 48 h following HI based on the infarct volume assay, and IgG extravasation into the brain tissue (**B**) was determined 24 h following HI by western blotting. *n* = 6–8. * *p* < 0.05, miR-210-LNA vs. Neg. Ctrl; ^#^ *p* < 0.05, HI vs. Sham; (**C**) Indication of pre-infarct area for serum IgG leakage imaging (blank square); Grey region represents the infarct core; and (**D**) Representative immunofluorescence images of IgG leakage in the cortex and tomato lectin infusion-labeled microvessels. Scale bar = 20 µm. In (**B**) and (**D**), Sham animals were subjected to right common carotid artery (CCA) exposure but without ligation and hypoxia.

To determine the effect of miR-210 inhibition on the BBB permeability after neonatal HI treatment, either miR-210-LNA or its negative control was delivered into the ipsilateral hemisphere of rat pups through i.c.v. injection 4 h after HI. Our results showed that miR-210-LNA treatment significantly reduced cerebral edema by about 50% at 48 h after HI (Figure 2A). Moreover, miR-210-LNA but not its negative control also significantly reduced the level of serum IgG in the brain tissue induced by HI insult (Figure 2B). To specify the region of BBB disruption, we performed IgG staining using brain slices from animals infused with DyLight 488 labeled *Lycopersicon esculentum* (Tomato) Lectin.

Immunofluorescence staining showed that IgG leakage observed in microvessels in the cortex neighboring the core infarct region (perilesional area), identified by cell loss and pyknotic nucleus, was reduced by miR-210-LNA at 24 h after HI (Figure 2C,D).

### 2.3. miR-210 Downregulates the Expression of Junction Proteins Occludin and β-Catenin

**Figure 3:**
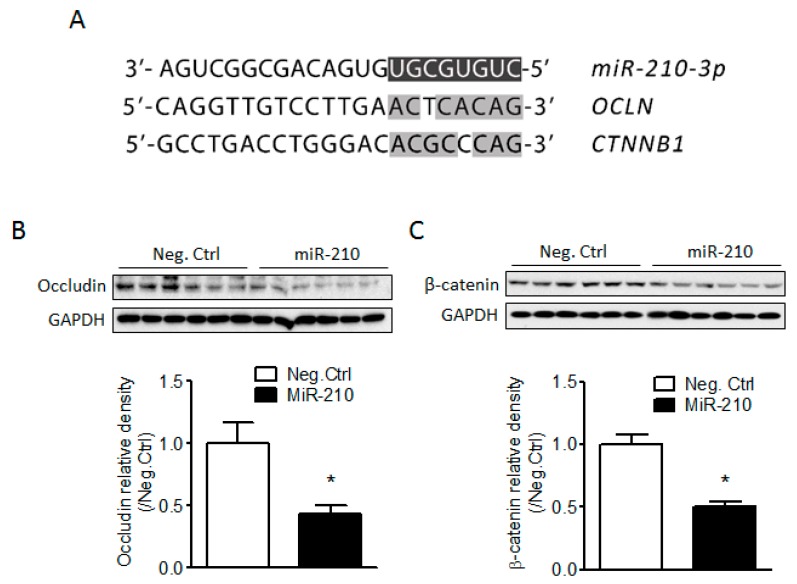
miR-210 downregulates the expression of junction proteins occludin and β-catenin in the brain of rat pups. (**A**) The diagram shows the 3′ UTRs of rat *OCLN* and *CTNNB1* transcripts are complementary to the seed sequence of rat miR-210. Pups received either miR-210 mimic or its negative control (Neg. Ctrl) via i.c.v. injection; The protein levels of occludin (**B**) and β-catenin (**C**) were determined 24 h following HI by western blotting. *n* = 6. * *p* < 0.05, miR-210 vs. Neg. Ctrl.

To identify potential target genes of miR-210, we analyzed the seed sequence of miR-210 (UGCGUGUC) and found two candidates, *OCLN* and *CTNNB1*, encoding tight junction (TJ) protein occludin and adherens junction (AJ) protein β-catenin, respectively, and both proteins are tightly associated with maintenance of the BBB integrity [14,36]. The transcripts of both genes contained potential miR-210 seed targeting sequences in their 3′ UTRs, as shown in Figure 3A. We then determined whether miR-210 is detrimental to BBB integrity by negatively regulating the expression of junction proteins. The result showed that miR-210 mimic significantly reduced the protein levels of occludin and β-catenin 48 h after injection as compared to its negative control (Figure 3B), suggesting that the effect of miR-210 on the increased vulnerability of BBB in response to neonatal HI is mediated by negative regulation of junction proteins occludin and β-catenin.

### 2.4. miR-210-LNA Treatment Protects Junction Proteins Occludin and β-Catenin from Neonatal HI Insult

**Figure 4:**
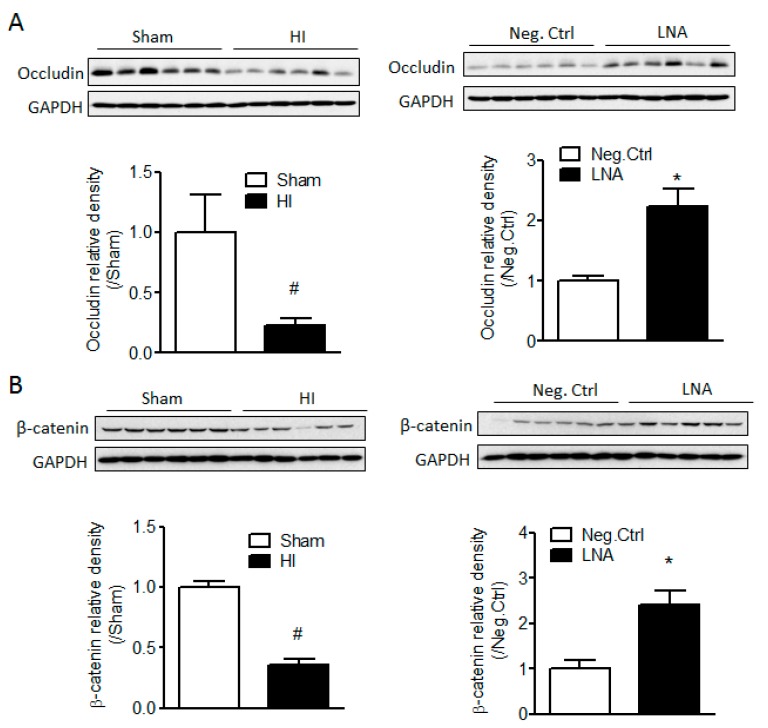
miR-210-LNA treatment protects junction proteins occludin and β-catenin from neonatal HI insult. Pups received either miR-210-LNA or negative control (Neg. Ctrl) via i.c.v. injection 4 h after HI insult. The protein levels of occludin (**A**) and β-catenin (**B**) were determined 24 h following HI by western blotting. *n* = 6. * *p* < 0.05, miR-210-LNA vs. Neg. Ctrl; ^#^ *p* < 0.05, HI vs. Sham.

To further investigate the role of endogenous miR-210 on the regulation of BBB junction proteins, we then tested whether miR-210 inhibition protected junction proteins occludin and β-catenin from neonatal HI insults. Our data showed that the protein abundance of occludin and β-catenin was significantly downregulated 24 h after neonatal HI, which was reversed by the miR-210-LNA treatment but not by its negative control (Figure 4).

## 3. Discussion

Neonatal HI insult not only induces periventricular white matter injury known as periventricular leukomalacia (PVL) and significant neuronal death [37,38], but also stimulates cerebral microvascular responses and BBB damage [7,15,16], ultimately leading to neurological functional deficits. The present study demonstrated that miR-210 was detrimental to the BBB permeability and cerebral edema formation after neonatal HI insult in rats, which was mediated by the negative regulation of BBB junction proteins occludin and β-catenin. Of importance, inhibition of miR-210 with miR-210-LNA significantly reduced cerebral edema, BBB leakage, and junction protein damage following HI insult, suggesting a novel mechanism of the neuroprotective effect of miR-210 inhibition in the neonatal HIE.

Our previous study demonstrated that miR-210 increased the vulnerability of the neonatal brain to HI insult, and miR-210 antagomir LNA treatment provided both short-term and long-term neuroprotection by the downregulation of brain miR-210 levels [33]. However, the mechanisms underpinning the detrimental effect of miR-210 in the neonatal brain remain unclear. Previous studies showed that miR-210 modulated endothelial cell response to hypoxia in vitro [30,31] and angiogenesis after ischemic stroke in animals [32]. We reported that brain miR-210 levels were significantly upregulated as early as 3 h after neonatal HI [33]. Moreover, in neonates, the brain microvascular responses and BBB damage occur rapidly and contribute significantly to brain injury after HI [15,16]. These findings implicate that miR-210 may play a critical role in BBB damage following neonatal HI insult. Thus, in the present study, we administered miR-210 mimic into the brain of neonatal rats and assessed the changes of BBB integrity in response to HI insult. We found that miR-210 exacerbated the vulnerability of the BBB in neonates to HI insult by increasing serum IgG extravasation into brain tissue and cerebral edema. Serum IgG is a widely used marker to assess the BBB permeability [16,18,39]. Muramatsu and colleagues detected the BBB permeability in postnatal day 7, 14, or 21 (P7, P14, or P21) rat pups after neonatal HI by assessment of IgG extravasation, and found that the barrier was more vulnerable to HI insult in rats at P7 compared to P21 rat pups [39]. A recent study from Ek and colleagues found that serum IgG leakage into the brain tissue was observed as early as 2 h and was dramatically increased at 24 h after HI insult in rat pups [16], which is in agreement with our result about serum IgG leakage in P7 rat pups. Moreover, we also found that miR-210 inhibition reduced IgG extravasation after neonatal HI. BBB opening/disruption results in the systemic leucocyte infiltration into the brain parenchyma and neuroinflammation, further enhancing cerebral edema formation [40]. Cerebral edema is a serious complication in both ischemic stroke in adult and neonatal HI brain injury, which leads to an increase in brain volume, elevation of intracranial pressure, brain herniation, and increased risk for hemorrhage from the damaged vessel, and finally causes cell death due to its compression effect on adjacent brain tissues [39,41,42,43,44]. Our data showed that miR-210 mimic significantly increased cerebral edema formation after neonatal HI, while miR-210-LNA significantly reduced cerebral edema caused by HI insult. Thus, the neuroprotection of miR-210-LNA may be attributed, in part, to its effect on the preservation of BBB integrity and the reduction of cerebral edema. Moreover, as we described, the development of cerebral edema starts very early after HI insult [11,12,45], which increases intracranial pressure and contributes to secondary brain tissue injury. Therefore, cerebral edema is not only one of the major consequences of HI, but also extends HI-induced brain injury. Based on this point, the reduction of brain edema by miR-210-LNA treatment not only diminished brain edema but also reduces other consequences resulting from brain edema such as cell death.

A common concept about BBB injury is that endothelial junction complexes undergo disruption, leading to increased paracellular permeability after hypoxic/ischemic insults [16,46]. Therefore, to understand the mechanisms of miR-210 on BBB integrity, we focused on the expression of junction proteins. By comparison of the seed sequence of rat miR-210 with the 3′ UTRs of the transcripts of junction proteins, we found that the seed sequences of miR-210 are complementary to the 3′ UTRs of the transcripts of *OCLN* and *CTNNB1*, encoding tight junction protein occludin and adherens junction protein β-catenin. BBB integrity is primarily determined by tight (TJs) and adherens junctions (AJs) between the cerebral endothelial cells [14,19]. Both junction complexes are constituted by multiple protein components. TJs at the BBB consist of transmembrane proteins occludin, claudins, junctional adhesion molecule (JAM)-1, and cytoplasmic zonula occludens protein-1 or -2 (ZO-1/2) [14,19,20]. AJs in the brain are composed of vascular endothelial cadherin (VE-cadherin), α-catenin, β-catenin, etc. [21,22]. The disruption of junction complexes results in increased permeability of the brain endothelial barrier in pathological conditions [16,23,24]. For instance, occludin is indispensable in the regulation of paracellular permeability of the BBB and related to the “tightness” of the TJs [47], and mutation of occludin gene leads to size-selective increases in the paracellular permeability of TJs [48]. The β-catenin, which forms a complex with VE cadherin and links directly to cytoskeleton [21,22], plays an important role in the stabilization of AJs [49]. The absence of β-catenin resulted in marked changes in actin cytoskeleton, a reduction of cell-cell adhesion strength, and an increase in paracellular permeability [50,51]. Moreover, β-catenin regulates the expression of other junction proteins. Conditional knockout of β-catenin gene from the adult brain endothelial cells induced BBB breakdown by downregulation of tight junction proteins [52,53]. Our data showed that both occludin and β-catenin protein levels were significantly reduced 48 h after miR-210 injection into the brain, and inhibition of miR-210 protected occludin and β-catenin proteins from neonatal HI, suggesting that the effect of miR-210 on the increased vulnerability of the BBB in response to neonatal HI is mediated by negative regulation of junction proteins occludin and β-catenin. Future study is needed to further identify whether miR-210 directly inhibit junction proteins using in vitro luciferase reporter gene assay. Moreover, the effect of miR-210 on BBB integrity will also need to be determined in an in vitro BBB model using brain microvascular endothelial cells.

It is a common notion that miRs regulate hundreds of genes in diverse biological pathways in response to stresses [54,55]. Therefore, in addition to junction proteins, miR-210 may orchestrate the BBB integrity by regulating other signaling pathways in response to neonatal HI. Glucocorticoid receptor (GR), which has been determined as a target of miR-210 in our previous study [33], may also be involved in BBB damage induced by neonatal HI. Indeed, it has been documented that glucocorticoids protect the BBB integrity from ischemic brain injury [35]. GR activation induced upregulation of occludin, ZO-1, or VE-cadherin [56,57], thus increasing transendothelial electrical resistance (TER) and improving BBB properties in a murine in vitro BBB model [58].

Moreover, in the present study, we revealed that miR-210 mimic was detrimental to BBB integrity in acute phase after HI. miR-210 mimic may produce a potential delayed effect on the neonatal brain by targeting junction proteins. Therefore, future studies may further investigate the potential long-term effect of miR-210 after neonatal HI brain injury. The small number of animals used for cerebral edema analysis was another limitation of this study. In conclusion, the main finding of this study is that miR-210 plays a detrimental role in BBB integrity by its negative regulation of junction proteins, thereby contributing to cerebral edema formation after neonatal HI. Of critical importance, the finding that the inhibition of miR-210 with miR-210-LNA significantly reduced BBB damage and cerebral edema formation following HI insult provides a proof of concept for a novel target of potential therapeutic intervention in the treatment of neonatal HI brain injury. This finding uncovers a new mechanism for the miR-210-LNA mediated neuroprotective effect on neonatal HI brain injury, which has been determined in our previous study [33]. Given the lack of effective therapy strategies against neonatal HIE, the present study deepens our understanding of the neuroprotective effect of miR-210-LNA and will be beneficial for the development of treatment strategies.

## 4. Materials and Methods

### 4.1. Hypoxic-Ischemia Model in Rat Pups

A modified Rice-Vannucci model was generated as previously described [33]. Briefly, postnatal 7-day-old (P7) rat pups (Charles River Laboratories, Portage, MI, USA) were fully anesthetized with inhalation of isoflurane (5% for induction and 2–3% for maintenance). The right common carotid artery (CCA) in the neck was exposed, double ligated with an 8.0 silk surgical suture, and then cut between two ligation sites. After surgery, pups were recuperated for 1 h at 37 °C, and then placed in a hypoxic incubator containing humidified 8% oxygen balanced with 92% nitrogen for indicated time at 37 °C. At the end of hypoxia, pups were returned to their dams for recovery. For the sham group, the right common carotid artery was exposed but without ligation and hypoxia. Rat pups of mixed males and females were randomly assigned into each experimental group. At least two litters were used for each experiment. The mortality rate of HI model is about 10%. The value of *n* means the number of rat pups used in each group. All procedures and protocols (approval number: 8150030, 06-Aug-2015) were approved by the Institutional Animal Care and Use Committee of Loma Linda University and followed the guidelines by the National Institutes of Health Guide for the Care and Use of Laboratory Animals.

### 4.2. Intracerebroventricular Injection (i.c.v.)

miR-210 mimic (Qiagen, Valencia, CA, USA), miR-210-LNA (Exiqon, Woburn, MA, USA), and their negative controls were prepared according to the manufacturers’ instructions, and were administered into the ipsilateral hemisphere of rat pups with a total volume of 2 µL via i.c.v. injection (coordinates: 2 mm posterior, 1.5 mm lateral, 3 mm below the skull surface) as we described previously [33]. In experimental protocol #1, pups were divided into two groups: (1) miR-210 mimic (60 pmol), and (2) miR-210 negative control (Neg. Ctrl; 60 pmol). The HI treatment was performed 48 h after the injection. In experimental protocol #2, pups were divided into four groups: (1) Sham, (2) HI, (3) HI + miR-210-LNA (50 pmol), and (4) HI + miR-210-LNA negative control (Neg. Ctrl; 50 pmol). Drugs were injected into the ipsilateral hemisphere 4 h after the HI treatment. The dose of miR-210 mimic or miR-210-LNA was determined in our previous study [33] whereby miR-210 increased the vulnerability of neonatal brain to HI insult, and miR-210-LNA provided neuroprotective effect with those doses. The purpose of miR-210 mimic injection prior to HI was to investigate the potential mechanism of miR-210 in the neonatal HI brain injury. To exclude the possibility that HI-induced brain injury concealed the detrimental effect of miR-210 to the neonatal brain, we conducted pretreatment with miR-210 mimic and detected changes of the vulnerability of neonatal brain to HI insult. Moreover, our previous [33] and present studies showed that miR-210 mimic pretreatment significantly reduced expressions of target genes 48 h after injection. In experiment 2, the purpose of posttreatment with miR-210-LNA was to reveal the potential clinic relevance of miR-210 inhibition on neonatal HI brain injury.

### 4.3. Brain Infarct Staining and Cerebral Edema Assay

Brain infarct was stained by 3,5-triphenyltetrazolium chloride monohydrate (TTC; Sigma-Aldrich, St. Louis, MO, USA) staining as we described previously [33]. Briefly, forty-eight hours after HI the rat pups were sedated with inhalation of isoflurane and brains were separated. Coronal slices of the brain (2 mm thickness, five slices per brain) were cut and immersed into a pre-warmed 2% TTC solution for 5 min at 37 °C. After washing with phosphate-buffered saline (PBS), slices were then fixed by 10% formaldehyde overnight. Both sides of each slice were photographed using a digital camera. Cerebral edema volumes were calculated by subtracting indirect from direct infarct volumes as previously described [34,35]. Direct and indirect infarct volumes were measured using National Institutes of Health (NIH) Image J software (Version 1.41, NIH: Bethesda, MD, USA). *V*_direct_ is the sum of the area of infarct (average of both sides) multiplying the thickness of each slice from five coronal slices. *V*_indirect_ is calculated by subtracting volume of normal area in the ipsilateral hemisphere from volume of contralateral hemisphere. Brain edema volumes were then calculated by subtracting indirect from direct infarct volumes and normalized to total brain volume.

### 4.4. Western Blotting

Western blotting was performed as we described previously [33]. Animals were anesthetized with inhalation of isoflurane and perfused with ice cold heparinized PBS to remove any residual blood in cerebral vessels. Then brain samples were collected and both ipsilateral and contralateral cerebrums were separated. Protein extraction was obtained by gentle homogenization in radioimmunoprecipitation assay (RIPA) lysis buffer (Santa Cruz Biotechnology, Dallas, TX, USA) with further centrifugation at 14,000× *g* at 4 °C for 20 min. The supernatant was collected, and protein concentration was determined using the BCA protein assay kit (Pierce, Rockford, IL, USA). Equal amounts of protein were separated by sodium dodecyl sulfate polyacrylamide gel electrophoresis (SDS-PAGE) and transferred to polyvinylidene fluoride (PVDF) membranes. Membranes were blocked and incubated with primary antibodies against occludin (Thermo Fisher, Waltham, MA, USA), β-catenin (Thermo Fisher), ZO-1 (Thermo Fisher), or Glyceraldehyde 3-phosphate dehydrogenase (GAPDH) (Abcam, Cambridge, MA, USA) overnight at 4 °C. Then membranes were incubated with secondary antibodies (Santa Cruz Biotech) for 1 h at room temperature. Immunoblots were then probed with enhanced chemiluminescence reagents (Pierce) and blots were exposed to Hyperfilm. The results were scanned and analyzed with NIH image J software. Protein levels were detected and normalized to GAPDH levels.

### 4.5. Labeling of Cerebral Microvessels In Vivo and Immunofluorescence Staining

Pups were anesthetized under isoflurane and cerebral vessels were labeled by transcardiac infusion of DyLight 488 labeled *Lycopersicon esculentum* (Tomato) Lectin (50 µL, Vector Lab, Burlingame, CA, USA) 5 min before sacrifice. The animals were then perfused with ice cold heparinized PBS to remove any residual blood in cerebral vessels followed by 4% paraformaldehyde (PFA). The brains were removed, post-fixed in PFA, and dehydrated in 30% sucrose for 2 days. After immersion in Optimal cutting temperature compound (OCT), brain tissues were sectioned at a thickness of 20 µm using a Leica cryostat (Leica, Buffalo Grove, IL, USA). Brain slices were blocked in 5% donkey serum (Jackson ImmunoResearch, West Grove, PA, USA) and then incubated with Alexa Fluor 594-conjugated donkey anti-rat IgG (H + L) secondary antibody (Thermo) for 1 h at room temperature. Brain slices were mounted and coverslipped using fluorescent mounting media (Dako, ‎Glostrup, Denmark). All slices were scanned with a Zeiss LSM 710 confocal microscopy (Zeiss, Oberkochen, Germany) in Advanced Imaging and Microscopy Facility of Center of Neonatal biology at Loma Linda University. Images were projected from 18 µm stacks using NIH Image J software.

### 4.6. Statistics

Data were expressed as mean ± standard error of the mean (SEM). Experimental number (*n*) represents neonates from different dams. Comparisons between two groups were analyzed using Student’s *t* test (unpaired, two-tailed) or non-parametric analysis, where appropriate. A *p*-value less than 0.05 was considered significant.
